# Association of 8-hydroxy-2’-deoxyguanosine with motoric cognitive risk in elderly Chinese people: RUGAO longevity and aging cross-sectional study

**DOI:** 10.1186/s12877-024-04943-0

**Published:** 2024-04-11

**Authors:** Qingqing Dai, Yajun Ma, Chang Liu, Ruixue Zhao, Qi Chen, Weijia Chen, Xiaofeng Wang, Xiaoyan Jiang, Shujuan Li

**Affiliations:** 1 Department of Geriatrics, School of Clinical Medicines, Beijing Tsinghua Changgung Hospital, Tsinghua University, Beijing, China; 2 Department of Neurology, FuWai Hospital, National Center for Cardiovascular Diseases, Peking Union Medical College, Chinese Academy of Medical Sciences, Beijing, 100037 China; 3 Department of Neurology, Beijing Chaoyang Hospital, Capital Medical University, Beijing, China; 4 Human Phenome Institute and National Clinical Research Center for Aging and Medicine , Huashan Hospital, Fudan University, Shanghai, China; 5 State Key Laboratory of Cardiology, Department of Pathology and Pathophysiology, School of Medicine, Tongji University, Shanghai, 200092 China; 6 Department of Neurobiology, School of Basic Medical Science, Capital Medical University, Beijing, 100069 China

**Keywords:** Motor cognitive risk syndrome, Longitudinal cohort study, 8-hydroxy-2’-deoxyguanosine, Risk factor

## Abstract

**Background:**

Motor cognitive risk syndrome (MCR) represents a critical pre-dementia and disability state characterized by a combination of objectively measured slow walking speed and subjective memory complaints (SMCs). This study aims to identify risk factors for MCR and investigate the relationship between plasma levels of 8-hydroxy-2’-deoxyguanosine (8-OHdG) and MCR among Chinese community-dwelling elderly populations.

**Methods:**

A total of 1312 participants were involved in this study based on the data of the Rugao Longevity and Aging Study (RuLAS). The MCR was characterized by SMCs and slow walking speed. The SCCs were defined as a positive answer to the question ‘Do you feel you have more problems with memory than most?’ in a 15-item Geriatric Depression Scale. Slow walking speed was determined by one standard deviation or more below the mean value of the patient’s age and gender group. The plasma of 8-OHdG were measured by a technician in the biochemistry laboratory of the Rugao People’s Hospital during the morning of the survey.

**Results:**

The prevalence of MCR was found to be 7.9%. After adjusting for covariates, significant associations with MCR were observed in older age (OR 1.057; *p* = 0.018), history of cerebrovascular disease (OR 2.155; *p* = 0.010), and elevated 8-OHdG levels (OR 1.007; *p* = 0.003).

**Conclusions:**

This study indicated the elevated plasma 8-OHdG is significantly associated with increased MCR risk in the elderly, suggesting its potential as a biomarker for early detection and intervention in MCR. This finding underscores the importance of monitoring oxidative DNA damage markers in predicting cognitive and motor function declines, offering new avenues for research and preventive strategies in aging populations.

## Background

Dementia is becoming a common problem among the geriatric population worldwide. A survey showed an estimate of the number of population with dementia in 2019 was 50 million, and a projection of 152 million could be reached in 2050 [1]. Motor cognitive risk syndrome (MCR) was firstly defined by Verghese in 2013 [2]. It is a pre-dementia and disability state combining objective slow walking speed and subjective memory complaints (SMCs). A worldwide epidemiological investigation, gathering data from 22 different cohorts across 17 nations, revealed a combined prevalence rate of 9.7%, with observed variations ranging from 2 to 16% among the cohorts [3]. Recent multicenter studies indicated that MCR is a motoric-based pre-dementia syndrome [4], and also a risk prediction of Vascular Dementia and Alzheimer Disease (AD) [5, 6]. It has been realized that risk factors of MCR could cover age, sex, level of education, obesity, low physical activity, depressive symptoms, and cardiovascular diseases [7]. A recent multicenter, nested case–control investigation into Alzheimer’s disease biomarkers among cognitively normal participants of the China Cognition and Aging Study in past 20 years. Findings showed that several biomarkers in the Alzheimer’s group began to deviate from the normal group years before diagnosis and cognitive decline became noticeable 6 years before diagnosis. Notably, as cognitive impairment worsened in the Alzheimer’s group, changes in CSF biomarker levels initially sped up before slowing down [8]. Therefore, analyzing the variation of biomarker in the different stages of cognitive impairment is very important for screening the diagnostic valuable biomarker.

Inflammation and oxidative stress mechanisms that co-occurred in many diseases were believed to be involved in these risk factors [9]. It was reported the cerebral small vessel disease, including the white matter hyperintensities and subcortical infarcts, were associated with inflammation which effecting the cognition and gait [10, 11]. A multicohort survey revealed a close relevance between MCR and higher tertiles of interlukin-6 (IL-6) and C-reactive protein (CRP) level [12]. Furthermore, it has been realized that vascular disease and Alzheimer Disease are correlated to both inflammation and oxidative stress [9]. Oxidative DNA damage has been determined as the key factor in blood–brain barrier breaking and neuronal degeneration [13], and 8-hydroxy-2’-deoxyguanosine (8-OHdG) could serve as a biomarker of oxidative DNV damage. The levels of 8-OHdG in tissue, blood, cerebrospinal fluid (CSF), and urine have been successfully determined by multiple researchers, and these studies have demonstrated the feasibility of employing 8-OHdG as a predictive biomarker of cardiovascular disease, post stroke depression, Parkinson’s disease, and Chronic Obstructive Pulmonary Disease [14–16]. It also further pointed out that the higher plasma levels of 8-OHdG in AD patients and DNA oxidation as a molecular pathway involved in early AD [17]. This study is among the first to investigate the plasma levels of 8-hydroxy-2'-deoxyguanosine (8-OHdG) as a biomarker for MCR, offering a novel approach to understanding and potentially mitigating the progression of pre-dementia conditions.

## Method

### Participants

This study was based on the 4 waves of the aging arm of the Rugao Longevity and Aging Study (RuLAS), a population-based observational two-arm cohort study conducted in Rugao, Jiangsu Province, China. We utilized stratified random sampling to ensure a representative sample of the elderly population in Rugao as previously described [18]. The study's objective is to collect data on aging-related phenotypes and risk factors to screen for and prevent MCR. The protocol of this study was reviewed and approved by the Human Ethics Committee of the Fudan University School of Life Sciences, approval number No.278. Written informed consent was obtained from all participants prior to the study.

The first wave survey (baseline) was conducted from November and December 2014, followed up by a second wave from April to June 2016, a third wave from November to December 2017, and a fourth wave from December 2019 to January 2020. In total, a number of 2201 participants were enrolled in the cohort. Inclusion criteria were complete cognition, walking capability, and independence in basic activities of daily living (BADL) which encompass functions such as micturition, defecation, grooming, toileting, eating, moving, walking, dressing, stair navigation, and bathing. The BADL were assessed by professionally trained researchers. Exclusion criteria included mental disorders, kidney disease, Parkinson's disease, vision loss, dementia, and significant cognitive impairment (i.e., a score ≤ 10 on the Revised Hasegama's Dementia Scale). Ultimately, 1312 people were participated in this study. Cerebrovascular disease, heart failure, and coronary artery disease were self-reported, which were diagnosed in the town-level or above hospital. Body Mass Index(BMI) was calculated by the formula: weight in kilograms divided by the square of height in meters (kg/m2). All the participants were assessed by 15-item Geriatric Depression Scale (GDS-15), and a score > 5 was defined as depression.

### Blood biomarker

Blood samples were collected from all participants by trained nurses in the morning before any food intake. Approximately 12 ml of blood was extracted from each individual for analysis [18]. The concentration of 8-OHdG (pg/ml) in the plasma was quantified by a skilled technician in the biochemistry laboratory of the Rugao People’s Hospital using a sensitive and specific enzyme-linked immunosorbent assay (ELISA).

### Motoric cognitive risk syndrome criteria

As mentioned previously, MCR is defined as a combination of SMCs and slow walking speed in older people without dementia or mobility disability [2]. The participants were interviewed by a question ‘Do you think you have more memory problems than most people?’ on the 15-item Geriatric Depression Scale [19]. If someone responded positively to the question, he/she was identified SMC. The scale was administered by trained researchers. On the other hand, walking speed was tested by walking 5 m at their usual pace without help and the result was calculated in meters per second. Consistent with previous studies, participants who were one standard deviation or more below the mean value for their age and gender group were considered to have a slow walking speed [20, 21]. The cutoff values of slow walking speed in this study are listed in Table 1.

**Table 1:** The cutoff values of slow walking speed

Age (years)	Male	Female
	**Walk Speed**	
≤ 74	≤ 0.78 m/s	≤ 0.72 m/s
75– 79	≤ 0.71 m/s	≤ 0.57 m/s
80–84	≤ 0.60 m/s	≤ 0.55 m/s
≥ 85	≤ 0.58 m/s	≤ 0.49 m/s

### Statistical analysis

We applied the Kolmogorov–Smirnov test to conduct normality tests for the characteristics of participants in the MCR and non-MCR groups. Continuous variables with a normal distribution are presented as the means ± standard deviations, and variables with a skewed distribution are expressed as medians (interquartile range). Categorical data are presented as frequency. For univariate analysis, differences in the clinical data were analysed by *t* -test and *χ*^2^ according to the data type. The risk factors for MCR were analyzed by binary logistic regression. Model 1 did not adjust for any confounding factors. Model 2 adjusted for age, cerebrovascular disease. Odds ratio (OR) and 95% confidence interval (95% CI) were reported. Analyses were performed using SPSS v22.0 software (SPSS Inc.). Statistical significance was set at *p* < 0.05 in all analyses.

## Result

In this study, we analyzed data from 1,312 participants, among which 103 individuals (7.9%) were identified with Motoric Cognitive Risk syndrome (MCR), while the remaining 1,209 participants (92.1%) were classified as non-MCR. The demographic and clinical characteristics of the study population are detailed in Table 2. The overall mean age of participants was 78.10 ± 4.45 years, and 628 (47.9%) participants were male. Within the MCR group, there was a higher proportion of females (60.2%) compared to males (39.8%). A majority of the participants, 1,045 individuals (79.6%), were farmers, and 66.4% were currently married. Lifestyle factors such as alcohol consumption and smoking were relatively low in this population, with 62.2% of participants reporting never alcohol consumption and 73.9% never smoking.

**Table 2:** Demographic and clinical data of participants' baseline characteristics

Variable	Total (*n* = 1312)	Non-MCR (*n* = 1209, 92.1	MCR (*n* = 103, 7.9%)	*p* value
Age, years	78.10 ± 4.45	78.01 ± 4.43	79.23 ± 4.54	0.007**
Sex, n				
Male	628 (47.9%)	587(48.6%)	41(39.8%)	0.088
Female	684 (52.1%)	622(51.4%)	62(60.2%)	
BMI, kg/m2	23.78 ± 3.70	23.80 ± 3.91	24.02 ± 3.81	0.575
Marital status, n				
Married	869(66.2%)	803(66.4%)	66(64.1%)	0.630
Unmarried	443(33.8%)	406(33.6%)	37(35.9%)	
Occupation, n				
Farmer	1045(79.6%)	956(79.1%)	89(86.4%)	0.076
Other	267(20.4%)	253(20.9%)	14(13.6%)	
Alcohol consumption, n				
Never				
Former	816(62.2%)	743(61.5%)	69(67.0%)	0.538
Current	101(7.7%)	99(8.2%)	7(6.8%)	
Smoking, n	395(30.1%)	367(33.4%)	27(26.2%)	
Never				
Former	969(73.9%)	882(73.0%)	83(80.6%)	0.218
Current	118(9.0%)	112(9.3%)	8(7.8%)	
Hypertension, n	225(17.1%)	215(17.9%)	12(11.7%)	
No				
Yes	850(64.8%)	780(64.5%)	70(68.0%)	0.482
Diabetes, n	462(35.2%)	429(35.5%)	33(32.0%)	
No				
Yes	1218(92.8%)	1120(92.6%)	98(95.1%)	0.344
Chronic pulmonary disease, n	94(7.2%)	89(7.4%)	5(4.9%)	
No				
Yes				
Coronary artery disease, n	1188(90.5%)	1096(90.7%)	92(89.3%)	0.657
No	124(9.50%)	113(9.30%)	11(10.7%)	
Yes				
Heart failure, n				
No	1189(90.7%)	1093(90.4%)	96(93.2%)	0.350
Yes	123(9.3%)	116(9.6%)	7(6.8%)	
Cerebrovascular disease, n				
No	1298(98.9%)	1196(98.9%)	102(99.0%)	0.921
Yes	14(1.1%)	13(1.1)	1(1.0%)	
Depression, n				
No				
Yes	1203(91.7%)	1116(92.3%)	87(84.5%)	0.006**
8-OHdG (pg/mL)	109(8.3%)	93(7.7%)	16(15.5%)	
	1223(93.2%)	1130(93.5%)	93(90.3%)	0.219
	89(6.8%)	79(6.5%)	10(9.7%)	
	184.40(147.65–222.00)	182.50(146.85–220.9	198.90(165.10–239.80)	0.002**

*Abbreviation*: *MCR* Motoric cognitive risk syndrome, *BMI* Body mass index, *8-OHdG* 8-hydroxy-2’-deoxyguanosine; *p < 0.05.; **p < 0.01

Health conditions were also reported, with a significant majority having no history of diabetes (92.8%), chronic pulmonary disease (90.5%), coronary artery disease (90.7%), cerebrovascular disease (91.7%), and heart failure (98.9%). Hypertension was absent in 64.8% of the participants, and 6.8% were identified as having depression. The depression participants accounted for 6.8%. The mean Body Mass Index (BMI) across all participants was 23.78 ± 3.70 kg/m^2^.

A key finding of our study was the difference in plasma 8-hydroxy-2’-deoxyguanosine (8-OHdG) levels between the MCR and non-MCR groups. The level of plasma 8-OHdG in the MCR group was was 198.90(165.10–239.80) pg/mL, significantly higher than 182.50(146.85–220.90) pg/mL observed in the non-MCR group (*p* = 0.002).

**Fig. 1:**
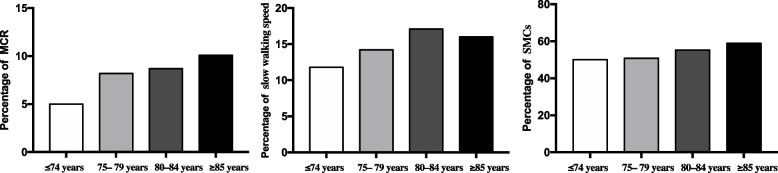
The percentage of MCR, slow walking speed and subjective cognitive complaints (SMCs) in different age groups

Further analysis (in Table 2) showed that older age, a history of cerebrovascular disease, and higher plasma levels of 8-OHdG were associated with an increased likelihood of being diagnosed with MCR. The rate of MCR with both SMCs and slow walking speed was 7.9%. Figure 1 provided the MCR rates in various age groups. It is obvious that the prevalence of MCR increases with age. The distribution of MCR rate was 5% (≤ 74 years), 8.2% (75–79 years), 8.7% (80–84 years), and 10.1% (≥ 85 years). The percentiles of slowing walking speed were 11.8% (≤ 74 years), 14.2% (75–79 years), 17.1% (80–84 years), and 16.0%(≥ 85 years), while that of SMCs were 50% (≤ 74 years), 50.8% (75–79 years), 55.2% (80–84 years), and 58.8%(≥ 85 years).

Table 3 summarized the results of logistic regression analyses for MCR using model 1, and it was found that age (OR 1.064, 95% CI 1.017–1.112; *p* = 0.007), cerebrovascular disease (OR 2.213, 95% CI 1.247–3.928;* p* = 0.007), and the level of plasma 8-OHdG (OR 1.007, 95% CI 1.003–1.012; *p* = 0.002) were dominant risk factors. For comparison, the logistic regression analyses were also conducted using model 2 by adjusting other covariates, and the results, as shown in Table 3, confirmed that the age (OR 1.057, 95% CI 1.010–1.106; *p* = 0.018), the history of cerebrovascular disease (OR 2.155, 95% CI 1.206–3.850; *p* = 0.010), and elevated level of plasma 8-OHdG (OR 1.007, 95% CI 1.002–1.012; *p* = 0.003) were still significantly associated with increased MCR risk.

**Table 3:** Logistic regression analysis of risk factors for motoric cognitive risk syndrome

Variable	Model 1		Model 2	
	**OR (95% CI)**	***P***	**OR (95% CI)**	***P***
Age, years	1.064(1.017, 1.112)	0.007**	1.057(1.010, 1.106)	0.018*
Sex, n	1.427(0.947, 2.151)	0.089	—	—
BMI, kg/m2	1.014(0.965,1.066)	0.574	—	—
Marital status, n	1.109(0.729, 1.687)	0.630	—	—
Occupation, n	0.594(.0333, 1.062)	0.079	—	—
Drinking, n	0.837(0.600, 1.166)	0.293	—	—
Smoking, n	0.781 (0.550, 1.110)	0.168	—	—
Hypertension, n	0.857(0.557, 1.318)	0.483	—	—
Diabetes, n	0.642(0.255, 1.618)	0.347	—	—
Chronic pulmonary disease, n	1.160(0.603, 2.232)	0.657	—	—
Coronary artery disease, n	0.687(0.312, 1.515)	0.352	—	—
Heart failure, n	0.902(0.117, 6.964)	0.921	—	—
Cerebrovascular disease, n	2.207(1.244, 3.916)	0.007**	2.155(1.206, 3.850)	0.010*
Depression, n	1.538(0.771, 3.069)	0.222	—	—
8-OHdG, (pg/mL)	1.007(1.003, 1.012)	0.002**	1.007(1.002, 1.012)	0.003**

Model 1: Original logistic regression model. *p < 0.05.; **p < 0.01

Model 2 was adjusted for age, cerebrovascular disease, *p < 0.05.; **p < 0.01

*Abbreviations*: *CI* Confidence interval, *OR* Odds ratio

## Discussion

Determination of the 8-OHdG level and demonstration of its correspondence to various diseases, such as stroke, Alzheimer's Disease, and depression, have been extensively realized in the literature [15, 17, 22]. In 1993 [23], 8-OHdG was first measured in three regions where an age group from 42 to 97 with cerebral cortex and cerebellum was screened. It was found that the level of 8-OHdG in mtDNA substantially raised 15-fold in the group more than 70 year-old. It seemed to suggest that the accumulation of oxidative damage to DNA in the human brain was age-related. The oxidative damage might impair the structures and functionalities of the cells, thus reduced mitochondrial activities. The damage was found in the brain and also in peripheral. It is therefore worthy to assess the treatment effectiveness with antioxidants, and Mecocci et al. provides a case study that has been applied to frailty and cognitive decline [24].

Numerous studies concur that the 8-OHdG plays a significant role in the mechanism of oxidation in AD patients. Pena-Bautista et al. analyzed and compared various oxidized products of proteins and DNA in the uring samples of AD patients and healthy controls. Statistically, significant difference was realized between the patient group and the control group [17]. Huang et al. identified the serum levels of adiponectin and 8-OHdG could act as specific and sensitive biomarkers for the early diagnosis and treatment of cognitive impairment in elderly diabetes mellitus type 2 patients. Similarly, a significant difference was observed in the serum levels of 8-OHdG and the scales of MMSE (*p* < 0.05) [25]. Another study from Moslemnezhad et al. also noted a remarkably higher plasma levels of 8-OHdG in the AD group compared to the control group, while the total antioxidant in the AD patients was significantly lower [26]. Recent study from Cao and Chen investigated the level of 8-OHdG in the mild cognitive impairment (MCI) with a participant pool, including AD and healthy control groups, of 352 individuals. The result showed that the level of 8-OHdG were highly expressed in MCI and AD groups. And the cognitive scale scores, including MoCA and MMSE scores, were negatively correlated with serum 8-OHdG in all three groups [27]. Our study provides the first population-based analysis linking plasma 8-OHdG levels with Motoric Cognitive Risk (MCR), investigating pre-MCI stages through slow walking speeds and subjective cognitive complaints among 1312 participants. The findings indicate a higher MCR prevalence in those with a history of cerebrovascular disease (14.7% vs 7.2%, *p* = 0.006) and in older participants (78.01 ± 4.43 VS 79.23 ± 4.54 years, *p* = 0.007), alongside higher plasma 8-OHdG levels (*p* = 0.002) in the MCR group..

As the first sign of degenerative or non-degenerative brain pathologies, declined gait speed may occur before other cognitive symptoms. Recent studies from Grande et al. and Meiner et al. suggested that the declined gait speed could be adopted to predict dementia [28, 29]. To maintain the stationary and dynamic balance, the visual, vestibular, proprioceptive components, motor and cognitive function were all important components [30]. The frontal subcortical circuits coordinate the functions of the motor, sensory, and cognitive networks so that to regulate the gait activity [31]. Beauchet et al. reported a GAIT study with a total of 171 individuals (28 MCR and 143 non-MCR). The result showed that the MCR- participants were related to smaller global and regional gray matter volumes involving premotor and prefrontal cortices; and it thus suggested that MCR may predict cortical neurodegenerative dementia [32]. So far, the molecular biological and genetic mechanisms for MCR were barely reported. Sathyan et al. conducted a 3-year follow-up study involving 530 individuals of 65 years and older. It indicated that single nucleotide polymorphisms in the transcriptional regions of cytokine interleukin 10 (IL-10) were associated with the incidence of MCR [33]. In another 36-week open-label clinical trial performed by Kumar et al., the results showed that the plasma concentrations of 8-OHdG were significantly higher in the older adults’ group by 348% compared to younger adults’ group. After providing antioxidant stress supplementation with GlyNAC (combination of glycine and N-acetylcysteine), the biomarkers of oxidant stress and 8-OHdG were significantly reduced [34].

In our study, it can be observed that 79.6% of the participants are farmers, while in other studies, the majority of the variables focus on educational level, with occupation being less frequently analyzed. The newly research, part of the China Cognition and Ageing Study, involved 29,072 participants suggests that maintaining a healthy lifestyle, including regular physical activity, can protect against memory deterioration in older age [35]. While, It's also important to consider the potential risks associated with farming, such as exposure to pesticides. A study published in the European Neurology journal in 2023 investigated the association between pesticide exposure and cognitive function in farmers. It focused on the effects of organophosphate and carbamate pesticides on cognitive performance, utilizing assessments such as the Mini Mental State Examination (MMSE) and Stroop Test. The findings revealed that long-term exposure to organophosphate pesticides could lead to lower cognitive function [35]. Unfortunately, direct comparisons with other studies are complicated by variations in study design, measurement tools, and the specific types of farming activities examined. Given the potential dual impact of farming on health outcomes, our study highlights the need for further research in this area. Future studies should aim to disentangle the protective versus detrimental effects of farming on physical and cognitive health.

### Strengths and limitations

One of the Strengths is the study benefits from a robust sample(1312 participants) that enhances the statistical power and generalizability of its findings within the context of the studied population. On the other hand, by examining the role of 8-OHdG, a marker of oxidative DNA damage, the study ventures into relatively unexplored territory in the context of MCR, offering potential new insights into its pathophysiology. Additionally, By highlighting modifiable risk factors such as cerebrovascular disease history alongside biomarker levels, the study underscores the potential for preventive healthcare measures to mitigate MCR risk. However, our study had several limitations. Firstly, the study is conducted in a specific geographic location (Rugao, China), which may limit the generalizability of the findings to other regions or ethnicities due to cultural, environmental, and genetic differences. Secondly, while the study adjusts for several covariates, there may still be unmeasured confounders that could influence the results, such as lifestyle factors, diet, or other environmental exposures. Thirdly, focusing solely on 8-OHdG may overlook the potential role of other biomarkers in MCR, which could provide a more comprehensive understanding of the syndrome's pathophysiology. The last but not the least, without longitudinal data, it's challenging to track the progression of MCR over time or determine the long-term impact of elevated 8-OHdG levels on cognitive and motor functions. Future longitudinal studies are necessary to establish temporal relationships and causality. Investigating the molecular and genetic mechanisms underlying the observed association could also provide valuable insights into potential therapeutic targets. Additionally, interventional studies examining the effect of antioxidants on 8-OHdG levels and cognitive outcomes in at-risk populations would be of great interest.

## Conclusion

In conclusion, our study identifies elevated plasma 8-OHdG levels as a significant biomarker associated with motoric cognitive risk in Chinese community-dwelling older adults. This association suggests that oxidative stress, as reflected by 8-OHdG, plays a crucial role in the early stages of cognitive decline. Our findings highlight the potential of 8-OHdG as a screening tool for identifying individuals at high risk of MCR, particularly among older adults with declined walking speed and subjective cognitive complaints. By advancing our understanding of the biomarkers associated with MCR, this study provide the new insight for research and preventive strategies in aging populations.

## Data Availability

The data that support the findings of this study are available from the corresponding author upon reason- able request.

## Abbreviations

MCR: Motor cognitive risk syndrome
8-OHdG: 8-Hydroxy-2’-deoxyguanosine
AD: Alzheimer disease
CSF: Cerebrospinal fluid
BADL: Basic activities of daily living
MCI: Mild cognitive impairment
